# The Role of Medications in Causing Dry Eye

**DOI:** 10.1155/2012/285851

**Published:** 2012-08-27

**Authors:** Frederick T. Fraunfelder, James J. Sciubba, William D. Mathers

**Affiliations:** ^1^Department of Ophthalmology, Casey Eye Institute, Oregon Health & Science University, 3375 SW Terwilliger BouLevard, Portland, OR 97239-4197, USA; ^2^The Milton J. Dance , Jr. Head & Neck Center, 6569 North Charles Street, Baltimore, MD 21204, USA

## Abstract

The purpose of this paper is to review the possible role of polypharmacy in causing dry eye disease (DED), reflecting the complex interactions and complications associated with the use of multiple systemic and topical ocular medications. The pharmacological, physiological, anatomical, and histological mechanisms causing dry mouth differ little from those causing dry eye. Oral polypharmacy is the most common cause of dry mouth, but has not been investigated as a cause of dry eye. Topical ocular polypharmacy has been shown to cause DED. Information on drugs that likely cause or aggravate DED and the controversial role of preservatives in topical ocular medications are examined. Systemic or topical ocular medications and preservatives used in topical ocular drugs may cause dry eye through the drug's therapeutic action, ocular surface effects, or preservatives, and the effects probably are additive. Long-term use of topical ocular medications, especially those containing preservatives such as BAK, may play an important role in DED and the role of polypharmacy needs further study. We review possible ways to decrease the risk of medication-related dry eye.

## 1. Introduction 

 In this paper, three researchers with different primary research interests (ocular toxicology FTF, dry mouth JJS, and dry eye WDM) examine several hypotheses concerning medications and their possible roles in causing DED. Hypothesis-driven studies are especially difficult for dry eye since diagnosis of this disease relies in part on subjective testing. In the future, perhaps testing such as osmolarity [1], standardized meibomian gland tests [2, 3], Fourier-domain optical coherence tomography [4], and so forth will be incorporated into clinical practice and will provide a greater degree of repeatability and predictability than is currently available. Until these become available, the studies cited here are heavily dependent on subjective assessments, case reports, and summaries of expert committees or organizations. The data presented here may be speculative and lacking quantification, but may be conceptually useful until a more evidence-based understanding of dry eye is achieved. This paper is also an attempt to give the clinician some guidelines to assist in their clinical decision making. 

## 2. Hypothesis 1: If a Systemic Medication Causes Dry Mouth, It May Also Cause or Aggravate Dry Eye

The similarity between keeping the eye and mouth moist is striking. The autonomic nervous system and nerve supply and function are nearly identical. The primary secretory glands of the eye are the main and accessory lacrimal glands, the meibomian glands, and other accessory glands. For the mouth the primary secretory glands are the parotid (serous secretion) and submandibular (mixed mucus and serous) with sublingual (mucus) and minor salivary glands (mucus). Both the mouth and eyes are supplied by acinar exocrine glands that produce proteins, electrolytes, and enzymes in an aqueous medium. Mucus secretions for the eye are primarily provided by the epithelial goblet cells of the conjunctiva and primarily the submandibular and 600 plus salivary glands of the mouth. Both the mouth and external eye are lined by stratified squamous epithelium [5, 6]. Pharmacologically both systems react in a very similar manner, with cholinergic and anticholinergic agents probably giving the most drying effects both for the eye and mouth (Table 1).

Of the top 100 best-selling systemic drugs in the United States in 2009, the Physician's Desk Reference mentioned the possibility of 22 of these drugs causing dry eye and dry mouth and another 34 causing only dry mouth [14]. Therefore, 56% of these drugs carry a possible sicca effect. Dry eye and dry mouth are primarily diagnosed by subjective complaints and are so recorded by pharmaceutical companies in Phase II and III studies. For dry mouth, the complaints are generally straightforward; subjects relate that they have a dry mouth or need increased fluids when they eat and often complain of difficulty in speech articulation [7]. On the other hand, dry eye may start with tearing and have complaints of burning, itching, foreign body sensation (sandy), mucus discharge, photophobia, and blurred vision and occasionally may start with tearing. The definition and testing for DED is still in transition. Currently it is defined as “a multifactorial disease of the tears and ocular surface that results in symptoms of discomfort, visual disturbance, and tear film instability with potential damage to the ocular surface. It is accompanied by increased osmolarity of the tear film and inflammation of the ocular surface” [15]. 

While those interested in clinical toxicology are aware that some drugs cause dry mouth, dry eye, and decreased sweating, to date there is no incidence data for drugs causing dry eye and/or dry mouth. We suggest that the drugs which have the most sicca effects probably affect both the eyes and mouth. 

As better tests become available, this hypothesis could be evaluated on a drug by drug basis.

We recommend that doctors ask their dry eye patients if any of their drugs caused a dry mouth as this may indicate that it also may be causing or aggravating their dry eye. This drug might then be substituted by another or given at a time when its peak drying effect would occur during sleep.

## 3. Hypothesis 2: Polypharmacy Plays a Role in the Causation or Aggravation of Dry Eye

 The most common definition of polypharmacy is the use of five or more prescription drugs. This is a problem mostly affecting the older population and continues to increase with age [16]. The National Ambulatory Medical Survey for person 60 years and older showed 12.4% used 2 prescription drugs daily, 27.3% used 3-4, and 36.7% used 5 or more [17]. This does not include the average of 2 or more over-the-counter medications (i.e., vitamins, aspirin, and decongestants) and then another 0.5 or more herbal preparations per day [18].

 Polypharmacy may be a problem because many medications interact with each other in ways that are difficult to predict and become increasingly complex as additional medications are added. There may also be a threshold effect for a particular mechanism or relating to a particular combination of drugs which then becomes clinically evident, whereas any single drug would not breach this threshold.

There is good evidence that polypharmacy is the primary cause of dry mouth. Prevalences are reported as high as 82% [19–22]. These rates increase with the addition of each known sicca-causing drug. The side effects of drugs, generally, have been reported to be 3 times greater in the older population [7, 23] which suffers most from dry eye. It is also rare that we understand most drug interactions involving more than 2 medications.

 A study by Schein et al. evaluating 2,481 individuals ages 65–84 years, found a strong correlation between the addition of each new class of drug known to have a sicca effect and an associated increased prevalence of dry eye and dry mouth [8]. The adjusted odds ratio was 1.8 (0.7–4.0) for one drug, 2.9 (1.2–6.9) for three drugs, and 7.0 (2.7–18.0) for five drugs, confirming the marked effect of adding known systemic drugs in causing dry eye and dry mouth.

 The importance of over-the-counter medications in polypharmacy as it pertains to the eye should not be overlooked. Both observational [11] and incidence-based epidemiologic studies [10] showed as little medication as a daily aspirin and/or multiple daily vitamins can cause dry eye. Possibly a multiple vitamin pill may be considered a polypharmacy in a single medication. 

 It is probable, that in many cases, more ocular drying occurs with advancing or multiple diseases, especially with such conditions as collagen vascular disease, regardless of medications. Increased disease severity usually leads to addition medications and higher doses. This may make dry eye causation difficult to determine as to disease, polypharmacy, or both. 

 Based on the above, we suggest that oral polypharmacy should itself be considered as a significant factor. Polypharmacy as a cause of dry eye has the potential to be even more important than with dry mouth since the risk of ocular sicca will be amplified by exposure to both topical and systemic medications.

## 4. Hypothesis 3: Systemic Medications May Induce Dry Eye

 There are few population-based observational studies examining systemic medications causing dry eye. This data has been variable and conflicting in part due to differing definitions and design. The Beaver Dam population-based, age-adjusted, cumulative 10-year dry eye study [10] examined 3,722 individuals. This represents the only-incidence based study tabulating dry eye that occurred only after drug exposure and thus more likely to be a causative agent. In a previous prevalence study, it was shown that both aspirin and antidepressants are risk factors causing dry eye. A recent cross-sectional survey on over 26,000 males over the age of 50 showed an increased prevalence of dry eye when treated with medications for depression and prostatic disease. This epidemiologic study showed antiandrogens as a cause of dry eye since the conjunctiva has *α*1 receptors [12] (Table 2).

 There is no population-based study regarding dry eye for any individual prescription drug, only for classes of drugs. Therefore, guidelines for any single medication are based on softer data as we are left with the literature, which is incomplete, and with postmarketing surveillance databases (FDA, WHO, and NR), which remain inadequate. Incomplete data and the difficulty of comparing data make it challenging to draw conclusions with regarding causality. The WHO classification system, Causality Assessment of Suspected Adverse Reactions, has definitions which include certain, probable/likely, possible, unlikely, unconditional/unclassified, and unassessable/unclassified categories [24]. This was developed for all of medicine and is the standard for the National Registry of Drug-Induced Ocular Side Effects as a guide to determine causality. The system relies on a clinical event, laboratory findings, or an identifiable pattern not explained by a concurrent disease, drug, or chemical and has a scientifically plausible mechanism for causation. Dechallenge (the response to withdrawal of the drug) and rechallenge (the response to the drug being reintroduced) data is weighted heavily. Based on this system, we have listed in Table 3 our assessment of which systemic drugs may cause or aggravate dry eye. 

 Systemic drugs may cause dry eye secondary to decreased tear production, alteration of nerve input including reflex secretion and decreased corneal sensation or a direct inflammatory effect on secretory glands [13]. Some may cause increased evaporation by changes in tear film composition, ocular surface abnormalities, number and quality of blinking, changes in mucus producing cells, and inflammatory changes in various ocular tissues. Of the 9 systemic drugs known to be secreted in the tears [13], 8 have been associated with causing dry eye. Rarely are drugs or its metabolites looked for in tears; however this may be an underappreciated as a cause or coincidental with ocular surface disease and DED. 

The role of systemic medication in DED is underappreciated, but available data to support this is still lacking since separating disease-induced DED from drug-induced DED is difficult.

## 5. Hypothesis 4: Topical Ocular Medications May Also Induce Dry Eye 

 Evaluation of topical ocular medications dry eye is more complicated than that with systemic drugs. Much of the data comes from trials primarily in young patients in phase 2 and 3 studies required for FDA approval. Dry eye side effects in these trials may be reported as various symptoms of dry eye without the patients saying that their eyes actually feel dry and therefore are not reported as such. There is a seldom objective sicca testing and therefore the term “dry eye” is not always mentioned in the Physician's Desk Reference. Evaporative dry eye is primarily of a set of symptoms associated with meibomian gland dysfunction leading to increased evaporation and osmolarity, in conjunction with ocular surface inflammation and possibly reduced lacrimal output. Many of the mechanisms whereby topical medications may cause dry eye differ little from systemic medications. However, the potential additional effects on evaporation from topical medication should increase the total incidence of dry eye. Complications from topical medications may result from their typically higher drug concentrations, greater frequency of application, preservative effects, higher incidence of drug-induced blepharitis, and drug-induced ocular surface inflammation [10–13]. All of these, either individually or combined, may result in tear film changes leading to increased evaporation and dry eye.

 Using the WHO Causality Classification System, we present our assessment of preservative-free topical ocular drugs which probably cause or aggravate dry eye (Table 4). Of the top 200 best selling drugs in 2009, surprisingly 7 are eye drop medications. These are latanoprost, cyclosporine, bimatoprost, moxifloxacin, travoprost, brimonidine, and olopatadine. Other than cyclosporine, all have shown probable ocular drying effects, although olopatadine (an antihistamine) may have marginal effects. The greatest potential for a topical ocular medication to have a drying effect may be with long-term therapy, polypharmacy, tear film, or corneal surface changes and especially drug-induced blepharitis. This potential is present with glaucoma therapy which has 40% of the 14+ billion dollar prescription topical ocular medication market. The importance of the number and frequency of each additional application or medication in causing a significant increase in ocular surface disease and dry eye are well documented [25, 26].

 Inflammation plays a significant and central role in the pathogenesis of dry eye and keratoconjunctivitis sicca [27]. Topical ocular medication can cause inflammation of ocular tissue, including uveitis and scleritis. However, the role of inflammation secondary to medication in causing dry eye is complicated and not fully understood. It is not clear whether these reactions produce allergic, toxic, or inflammatory side effects. Many mechanisms are possible since medications may interfere with goblet cells, meibomian glands, ocular surfaces of the conjunctiva and cornea, and poorly understood tear film properties. As pointed out by Baudouin et al. [28, 29], the complexity of inflammation occurring on the ocular surface may be in part due to medications. Recently a report of probable glaucoma medication-(with preservative) induced meibomian gland dysfunction was associated with a 50% incidence of dry eye; however, the patient had the signs of dry eye but did not have the symptoms [30]. This is not surprising since there may be a poor correlation between objective and subjective signs of dry eye [31].

 With use of each additional known sicca-causing systemic class of drugs, ocular sicca complaints were also increased [32]. The same is true for each additional topical ocular medication [33]. What is not known is whether oral plus topical ocular dry-eye-producing agents are additive since the study has not been done.

 There is evidence that topical ocular drugs, including topical preservatives, may cause ocular surface inflammation and trigger dry eye and these effects are likely to be additive to the sicca effects of systemic drugs. This potential is probably under-appreciated by the clinician. Many commercial chronic topical ocular medications can cause changes in the ocular tear film and/or blepharitis (Table 4). 

## 6. Hypothesis 5: Long-Term Topical Ocular Medications with the Preservative, Benzalkonium Chloride (BAK), Can Cause Dry Eye 

 There is disagreement as to whether or not long-term BAK can cause dry eye. Some feel that changes described from long-term BAK use can be normal aging changes or not due to the preservative and they ask for better designed, long-term studies. There is no doubt that topical preservatives can cause ocular surface toxicity, which may take a number of forms, one of which includes DED.

 There have been randomized, controlled multicenter, double-masked, prospective parallel group studies for 6 months with 605 patients comparing travoprost 0.0015 and 0.004% [34], bimatoprost, and timolol in 379 patients upto 1 year [35] and latanoprost in 829 patients up to 2 years [36]. Travoprost contains an ionic buffering system as a preservative, bimatoprost contains BAK 0.05 mg/mL, latanoprost 0.02% BAK, and timolol 0.01% BAK. Frequency application was 1-2 times per day. None of these studies showed significant anterior segment pathology which could cause dry eye.

 Baudouin et al., recently reviewed their extensive experience and the literature from over the past 20 years, which showed that topical ocular mediations containing BAK caused tear film instability, ocular surface changes, conjunctival inflammation, epithelial apoptosis, and subconjunctival fibrosis [37]. There are 170 papers (Medline) that pertain to BAK effects on the eye, amplifying the experience of Baudouin and colleagues.

 A study of 9,658 patients, using objective and subjective criteria, found significantly more signs and symptoms of ocular surface disease in patients taking glaucoma medications with preservatives (95+% using BAK) than those using preservative-free formulations. Dry eye sensation was reported as twice as common, foreign body sensation three times, and stinging and burning two and a half times as common in the preservative group as compared to the preservative-free group. The incidence of anterior and posterior blepharitis was about 3 times more common with glaucoma medications with preservatives. All of the above findings were highly significant and had *P* < 0.0001 values [38].

 Rossi et al., using the definition of dry eye as the presence of superficial punctuate keratitis and decreased fluorescein break-up time, showed a higher incidence of dry eye in patients taking topical ocular medication containing BAK than controls (*P* < 0.01) [26]. Dry eye was found in 40% of those taking 2-3 drops per day compared to 11% once a day and 5% for no eye drops.

 Leung et al., using the Ocular Surface Disease Index (OSDI) for measuring the symptoms of dry eye, show a 59% prevalence rate of dry eye in at least one eye in patients on topical ocular glaucoma medications with BAK compared to their overall prevalence rate of 27% in non-glaucoma patients [33]. In glaucoma patients on topical ocular medications, there was an abnormal Schirmer's result in 61% of cases, lissamine green staining in 22%, decreased tear film break-up times in 78% and decreased tears in 65%. Each additional BAK containing eye drop was associated with 2 times higher odds of showing abnormal results on lissamine green staining test (*P* < 0.034).

 Fechtner et al., using the OSDI for measuring dry eye symptoms, showed that scores significantly worsen when comparing patients on a single topical ocular glaucoma medication as to those on 2 medications (*P* < 0.007) or 3 medications (*P* < 0.0001) [25].

 In these 3 studies (Fechtner, Leung, and Rossi), patients were not stratified as to length of time on the drug [25, 26, 33]. Jaenen et al. discovered marked differences between topical ocular medications with and without preservatives while the study by Leung et al. showed the impact of a single drop containing BAK which may speak for more of drying effect of BAK compared to the chronic exposure to the drug itself [33, 38]. Glaucoma may require topical ocular medications for decades. Since most commercial medications contain BAK this masks preservative versus drug influence as to causation of dry eye. There are, however, numerous reports placing heavier emphasis on BAK [25, 26, 33, 37, 38]. 

Commercial preparations containing BAK have caused irreversible ocular damage in other studies. Broadway et al. studied 124 patients and found that long-term (over 3 years) combination topical ocular glaucoma therapy was a significant risk factor for trabeculectomy surgery failure [39]. Failure was associated with statistically significant more inflammatory cells in the superficial and deep substantia propria. Preoperative subclinical inflammation induced by previous exposure to long-term topical ocular glaucoma medication was felt to be the cause of this inflammation and higher surgical failure rate. Punctal stenosis is not uncommon from chronic exposure to topical ocular medication [40, 41]. In the NR, there are numerous cases of unilateral punctual stenosis occurring only on the side of the patient's monocular glaucoma.

 Schwab et al. showed long-term glaucoma medication with preservatives caused conjunctival foreshortening with shrinkage including conjunctival scarring [42]. This was confirmed by Thorne et al. in a series of 145 cases in which 97.4% of these patients had antiglaucoma medication [43].

 The standard of proof demanded by the FDA and International Dry Eye Workshop implicating BAK and dry eye has not been met. In our opinion it is in large part because the long-term studies that are necessary are extremely difficult and expensive. The marketplace has acted to some degree already, with 10–15% of artificial tears being sold are preservative free, not including non-BAK preservatives. There is good evidence to show that topical BAK may cause dry eye. We suggest, that in addition, this action may interact synergistically with the effects of polypharmacy or with a previously compromised ocular surface. 

## 7. Hypothesis 6: Medication-Induced Dry Eye and Dry Mouth May Masquerade as Primary Sjögren's Syndrome

Since so many classes of drugs cause both a dry mouth and dry eye (Table 1) and since polypharmacy is the number one cause of dry mouth and possibly important in dry eye, the effects of polypharmacy must be ruled out before diagnosing primary Sjögren's syndrome. In a dry eye and mouth study which showed that the addition of each class of drugs increased the prevalence of dry eye or dry mouth, the authors also found that if a female took 7 prescription drugs with known sicca effects on the mouth or eyes, there was also an increased prevalence of vaginal dryness [8]. 

In evaluating patients for primary Sjögren's syndrome, a history of polypharmacy and recent drug use must be sought. Any changes in medication, especially during the previous 3 months, should be considered as potentially causing or worsening dry mouth or dry eye. Labeling a patient as having primary Sjögren's without unequivocal serologic and tissue studies (minor salivary gland biopsy) may add significant emotional stress resulting from the perceived implications of an autoimmune disease diagnosis.

## 8. Conclusion 

 The role of medications in causing or aggravating DED is complex and this paper suffers from oversimplification and incomplete data. Nonetheless, sufficient information is available to make the following recommendations to clinicians.

1. The role of systemic and topical ocular medications in causing dry eye is probably underappreciated. There is a need to determine if the drying effects of systemic and topical ocular medications are additive or synergistic.
2. Oral polypharmacy needs to be studied as a cause of dry eye. The total number of drugs should include not only prescription drugs, but topical ocular medications, over-the-counter medications, and herbals as well.
3. Clinicians should remember that if a medication causes a dry mouth, it may also cause or aggravate dry eye.
4. It is probable that the duration of topical ocular therapy is relevant as a cause of dry eye. Topical BAK may be the primary factor in causing DED and ocular surface disease in a given patient.
5. It is important to question the diagnosis of primary Sjögren's Syndrome without adequate laboratory confirmation if the patient has a history of polypharmacy or if onset of dry eye or mouth occurred within 3 months of a change in medication.
6. Available on the National Registry of Drug-Induced Ocular Side Effects website, http://www.eyedrugregistry.com/, are
  - bibliography of a drug's side effect profile listed in Tables 3 and 4;
  - list of drugs causing dry mouth;
  - causes of polypharmacy.

## Figures and Tables

**Table 1:** Systemic medications that may cause both dry mouth and dry eyes [7–9].

Class of drugs	
Adjuncts to anesthesia	Antipyretic agents
Analgesics	Antirheumatic agents
Antiandrogens	Antispasmodics
Antiarrhythmics	Antivirals
Anticholinergics	Anxiolytics
Antidepressants	Bronchodilators
Antiemetics	Chelating agents
Antihistamines	Decongestants
Antihypertensives	Diuretics
Antileprosy agents	Neurotoxins
Antimalarial agents	Opioids
Antimuscarinics	Psychedelic agents
Antineoplastics	Retinoids
Antiparkinsonians	Sedatives and hypnotics
Antipsychotics	

**Table:** (a)

Drug	Relative risk	*P* value
Antihistamine use	1.24	0.05
Antianxiety drug use	1.43	0.02
Antidepressant use	1.44	0.03
Diuretic use	1.18	0.06
Oral steroid use	1.43	0.01
Vitamin use	1.30	0.02

**Table:** (b)

Drug	Odds ratio	95% confidence interval
Antiandrogen*	1.35	1.01–1.80
Antidepressants*	1.90	1.39–2.61
Aspirin**		
(1/day)	1.07	0.69–1.67
(≥2/day)	1.98	1.36–2.89

**Table 3:** Systemic drugs probably causing or aggravating dry eyes [13].

Class	Examples
Antihypertensive agents (beta-agonists)	Acebutolol
Antihypertensive agent (alpha-agonists)	Atenolol
Antiarrhythmic agents (beta blockers)	Carvedilol
	Labetalol Metoprolol Nadolol Pindolol Clonidine Prazosin Oxprenolol Propranolol

Antipsychotic agents	Chlorpromazine
	Fluphenazine Lithium carbonate Perphenazine Prochlorperazine Promethazine Quetiapine Thiethylperazine Thioridazine Brompheniramine Carbinoxamine Chlorphenamine (chlorpheniramine) Clemastine Cyproheptadine Dexchlorpheniramine

Bronchodilators	Diphenhydramine
Antispasmodics/ antimuscarinic	Doxylamine
Antiarrythmic agents	Ipratropium
	Atropine Homatropine Tolterodine Hyoscine (scopolamine) Hyoscine methobromide (methscopolamine) Disopyramide

Antineoplastic agents	Busulfan
	Cyclophosphamide Interferon (alpha, beta, gamma, or PEG) Vinblastine Cetuximab Erlotinib Gefitinib

Antihistamines	Cetirizine
	Desloratadine Fexofenadine Loratadine Olopatadine Tripelennamine

Antidepressants	Citalopram
	Fluoxetine Fluvoxamine Paroxetine Sertraline

Antileprosy agents	Clofazimine

Antirheumatic agents/analgesics	Aspirin
	Ibuprofen

Sedatives and hypnotics	Primidone

Drugs secreted in tears	Aspirin
	Chloroquine Clofazimine Docetaxel Ethyl alcohol Hydroxychloroquine Ibuprofen Isotretinoin

Antiandrogens	Tamsulosin
	Terazosin Doxazosin Alfuzosin

Neurotoxins	Botulinum A or B toxin

Antimalarial agents	Chloroquine
	Hydroxychloroquine

Retinoids	Isotretinoin

Antiviral	Aciclovir

Thiazides	Bendroflumethiazide
	Chlorothiazide Chlortalidone Hydrochlorothiazide Hydroflumethiazide Indapamide Methyclothiazide Metolazone Polythiazide Trichlormethiazide

Cannabinoids	Dronabinol
	Hashish Marijuana

Chelating agents	Methoxsalen

Strong analgesics	Morphine
	Opium/opioids

Antipsychotic agents	Pimozide

*References for the above systemic medications provided at http://www.eyedrugregistry.com/.

**Table 4:** Topical ocular drugs that may cause or aggravate dry eye [13].

Class	Examples
Agents used to treat glaucoma	
Beta-blocking agents	Betaxolol Carteolol Levobunolol Metipranolol Timolol
Adrenergic agonist drugs	Apraclonidine Brimonidine
Carbonic anhydrase inhibitors	Brinzolamide Dorzolamide
Cholinergic agents	Pilocarpine
Prostaglandins	Bimatoprost Latanoprost Travoprost
	Dipivefrine Unoprostone Ecothiopate

Agents used to treat allergies	Emedastine Olopatadine

Antiviral agents	Aciclovir Idoxuridine Trifluridine

Decongestants	Naphazoline Tetryzoline

Miotics	Dapiprazole

Mydriatics and cycloplegics	Cyclopentolate Tropicamide Hydroxyamfetamine

Preservatives	Benzalkonium chloride

Topical local anesthetics	Cocaine Proxymetacaine Tetracaine

Topical ocular NSAIDs	Bromfenac Diclofenac Ketorolac Nepafenac
