# Corrigendum to “The Role of Medications in Causing Dry Eye”

**DOI:** 10.1155/2019/2989680

**Published:** 2019-03-04

**Authors:** Frederick T. Fraunfelder, James J. Sciubba, William D. Mathers

**Affiliations:** ^1^Department of Ophthalmology, Casey Eye Institute, Oregon Health & Science University, 3375 SW Terwilliger BouLevard, Portland, OR 97239-4197, USA; ^2^The Milton J. Dance, Jr. Head & Neck Center, 6569 North Charles Street, Baltimore, MD 21204, USA

In the article titled “The Role of Medications in Causing Dry Eye” [1], there were errors in Tables 3 and 4, where many of the drugs are misclassified and/or allocated to incorrect therapeutic drug classes. This was raised by Nardine Nakhla and Rosemary Killeen [2]. The parts that should be corrected in Tables 3 and 4 are as follows:

## Figures and Tables

**Table 3:** Systemic drugs probably causing or aggravating dry eyes.

Drug name	Actual drug class
Acebutolol	Beta-blocker with intrinsic sympathomimetic activity
Alfuzosin	Alpha_1_-blocker
Atenolol	Beta-blocker
Atropine	Anticholinergic; ophthalmic agent, mydriatic
Brompheniramine	Histamine H_1_-antagonist, first generation
Chlorpheniramine	Histamine H_1_-antagonist, first generation
Chlorthalidone	Diuretic, thiazide-related
Clemastine	Histamine H_1_-antagonist, first generation
Clofazimine	Antibiotic
Clonidine	Alpha_2_-adrenergic agonist
Cyproheptadine	Histamine H_1_-antagonist, first generation
Dexchlorpheniramine	Histamine H_1_-antagonist, first generation
Diphenhydramine	Histamine H_1_-antagonist, first generation
Doxazosin	Alpha_1_-blocker
Doxylamine	Histamine H_1_-antagonist, first generation; ethanolamine derivative
Dronabinol	Antiemetic; appetite stimulant
Homatropine	Anticholinergic; ophthalmic agent, mydriatic
Hyoscine (scopolamine)	Anticholinergic; ophthalmic agent, mydriatic
Hyoscine methobromide	Antimuscarinic/antispasmodic
Indapamide	Diuretic, thiazide-related
Ipratropium	Anticholinergic
Metolazone	Diuretic, thiazide-related
Prazosin	Alpha_1_-blocker
Primidone	Anticonvulsant; barbiturate
Promethazine	Histamine H_1_-antagonist, first generation
Tamsulosin	Alpha_1_-blocker
Terazosin	Alpha_1_-blocker
Tolterodine	Anticholinergic

**Table 4:** Topical ocular drugs that may cause or aggravate dry eye.

Drug name	Actual drug class
Apraclonidine	Alpha_2_-agonist
Brimonidine (ophthalmic)	Alpha_2_-agonist; antiglaucoma
Dapiprazole	Alpha-blocker
Dipivefrine	Prodrug of epinephrine
Naphazoline (ophthalmic)	Alpha_1_-agonist; vasoconstrictor; imidazoline derivative
Tetryzoline (ophthalmic)	Adrenergic agonist; vasoconstrictor; imidazoline derivative
